# An Algorithm for Calculating the Probability of Classes of Data Patterns on a Genealogy

**DOI:** 10.1371/4fd1286980c08

**Published:** 2012-12-14

**Authors:** Jordan M. Koch, Mark T. Holder

**Affiliations:** University of Kansas; Department of Ecology and Evolutionary Biology, University of Kansas

## Abstract

Felsenstein's pruning algorithm allows one to calculate the probability of any particular data pattern arising on a phylogeny given a model of character evolution. Here we present a similar dynamic programming algorithm. Our algorithm treats the tree and model as known. The algorithm makes it feasible to calculate the probability that a randomly selected character will be a member of a particular class of character patterns. Specifically, we are interested in binning patterns by the number of parsimony steps and the set of states observed at the tips of the tree. This algorithm was developed to expand the range of data set sizes that can be used with Waddell et al.'s marginal testing approach for assessing the adequacy of a model. The algorithms introduced can also be used in likelihood calculations which correct for ascertainment biases. For example, Lewis introduced an Mkv model which corrects for the lack of constant sites. The probability of a constant pattern arising can be calculated using the algorithm that we present, or by enumerating all possible constant patterns and calculating the probability of each one. Because the number of constant data patterns is small, both methods are efficient. However, elaborations of the Mkv model (such as those in Nylander et al) require calculating the probability of parsimony-uninformative patterns arising. For large trees and characters with many possible character states, the number of possible parismony-uninformative patterns is immense. In these cases, the algorithms introduced here will be more efficient. The algorithm has been implemented in open source software written in C++.

## Background

Conducting likelihood-based phylogenetic inference requires calculating the probability that a particular set of characters would arise under the assumption that the evolutionary process is described by a combination of tree topology, branch lengths, and numerical parameters for a model of character evolution. In a landmark paper^1^ , Felsenstein introduced a dynamic programming algorithm, the pruning algorithm, which allows one to perform this set of probability calculations efficiently for a discrete-state character. Felsenstein's algorithm sweeps down the tree once, making its computational complexity linear with respect to *N*, the number of tips in the tree. At each internal node that is the parent of another internal node, it must consider the transition probabilities between all possible pairs of unseen states. Thus the algorithm scales with the square of the number of character states, *K*. The number of possible ancestral character state combinations that could result in any pattern is on the order of *K^(N-^* ^2^ *^)^*, but the pruning algorithm enables the probability of the pattern to be calculated in a number of steps that scales on the order *(N-2)K^2^*.

In some contexts, we would like to be able to calculate the probability that any member of a class of patterns would arise on a tree. For example, Waddell et al.^2^ introduced a method for assessing the adequacy of a substitution model in phylogenetics. They noted that tests of model adequacy introduced by Reeves^3^ and Goldman^4^ ^5^ often lack power, particularly for data sets with a large number of sequences. These tests use a likelihood-ratio test statistic to compare the probability of the data under a phylogenetic model to the probability of the data under an "unconstrained", multinomial model. The multinomial model has a free parameter for every possible data pattern. The likelihood under this unconstrained model is an upper bound on the likelihood for any independent-sites model^4^ because the unconstrained model can perfectly match the relative frequency of every observed pattern. In these tests, the inherent lack of power arises from the enormous number of free parameters in the multinomial model. The number of possible patterns grows exponentially with the number of tips in the tree. Because each of the *N* leaves can assume any of the *K* states, there are *K^N^* possible patterns. The multinomial model makes no constraint on the expected frequencies (other than that they sum to 1), so there are *K^N^*-1 free parameters in the model. For the test to detect that the phylogenetic model is inadequate, the likelihood improvement associated with the unconstrained model must be large enough to overcome the substantial penalty for overparameterization that comes with this very large number of free parameters.

Waddell *et al*.^2^ provide a more powerful test using a likelihood ratio, binning the data patterns into groups of similar characters. They suggest grouping the characters into bins based on the observed number of steps (according to the parsimony criterion) and the set of states that were observed. A well-constructed marginal test, such as their test, can detect deficiencies in the model caused by underestimating certain aspects of the process of molecular evolution. For example, if a particular amino acid is required at a site in a protein-coding sequence, then the third base position of the codon may be constrained to be a purine. Over long periods of evolution, sites will exhibit a large number of substititions, but only two states (A or G). An *iid* (independent, identically-distributed) model of nucleotide change will consistently underpredict the prevalence of such patterns. By binning all patterns that display only A and G and that imply 9 steps according to parsimony (for example), Waddell **et** *al* 2's marginal test reveals the repeated under-prediction of this class of data patterns by an *iid* model. Importantly, the test can do this without introducing a large number of free parameters in the multinomial model that provides the reference likelihood. This results in a more powerful test. To calculate the probability of any member of a class of patterns arising on a tree, Waddell **et** *al^2^*simulated a large number of characters and counted the proportion of them which displayed one of the patterns in the class. This simulation-based approximation clearly does not scale to large trees. The algorithm that we introduce here will enable the relatively efficient calculation of the probability of a class of data patterns, thus making the marginal tests of Waddell *et* *al* *.* 2 available for a larger range of phylogenetic problems.

The algorithm presented below is a dynamic programming approach to calculating the probability of a data pattern belonging to a class of patterns. Specifically, these classes of patterns all share the same set of observed states, the number of steps according to parsimony, and downpass state set according to the Fitch^6^ algorithm. The probabilities used in the marginal test of Waddell *et al.* 2 can be obtained from these probabilities by summing over all possible downpass state sets. When referring to "the Fitch algorithm" below, we refer to the "preliminary phase" (commonly referred to as the "downpass") of identifying possible ancestral states in the terminology of Fitch^6^ . This part of the parsimony reconstruction algorithm was originally published in Fitch^7^ . It allows one to calculate the parsimony score of an unordered character in a single pass down the tree. At each internal node, the algorithm composes a set of states. This state set, referred to as the downpass state set, is not the set of possible states in the most parsimonious reconstruction. It is only the preliminary phase of creating the most parsimonious reconstruction. Nevertheless, it is useful because when we encounter an internal node in Fitch's downpass, the only pieces of necessary information are the downpass state sets of the node's children and the minimal number of parsimony steps accrued in the subtrees rooted at each child. Specifically, the downpass starts by initializing the leaves of the tree such that a leaf's downpass state set is identical to the set of states observed for that taxon and the parsimony score accrued is 0. Let *D_n_* represent the downpass state set of a node and *S_n_* denote the minimal number of parsimony steps contributed by the subtree rooted at node *n*. *A(n)* denotes the first child of node *n* and *B(n)* denotes the second child. The algorithms described are restricted to fully resolved trees. Because branch rotation is not significant in phylogenetics, the designation of which child is the "first" and which is the "second" is arbitrary. The downpass algorithm of Fitch is performed as a postorder traversal, and at an internal node *n*:



The dynamic algorithm described below relies on the fact that we can pre-calculate all of the possible downpass state sets, and all of the combinations of child nodes' downpass state sets that could result in these state sets.

## Description of the algorithm

The algorithm proceeds by calculating the probability of generating different classes of patterns for the subtree rooted at a node. For the subtree rooted at node *n*, let *Q_s,t,d,a_(n)* denote the probability of generating a specific class of patterns conditional on an ancestral state, where *s* denotes the number of parsimony steps in the subtree, *t *denotes the set of states being observed at the tips of the subtree, *d* is the downpass state set of node *n*, and *a* denotes the character state that for node *n*. Thus, *Q_s,t,d,a_(n)* is the probability of generating any pattern that displays *s* steps, the states *t*, and a downpass of *d* in the subtree given that state *a* was the ancestral state at node *n*.

The algorithm will sweep over the tree in postorder traversal (leaves to root), and fill in a lookup table at each node to hold these probabilities. Let *S* denote the set of all of the states; for a DNA sequence matrix, *S*={A,C,G,T}. Note that the first subscript of *Q* is a non-negative integer that cannot exceed the maximum possible parsimony score. The second subscript, *t*, (the set of observed states) indexes each possible set of observed states. This is the power set of *S* with the empty set excluded. We do not consider missing data, and therefore do not need to consider the possibility that no states will be observed in a subtree. We will denote the power set of *S* as *Y(n)* and the power set of *S* with the empty set excluded as *Z(S).* The size of Z(S) is 2^|*S*|^-1. The third subscript, *d*, indexes the power set of the observed state set. Once again the empty set is excluded from this power set, because the downpass state set in Fitch's algorithm is never empty. Because a state must be observed in a leaf of the subtree for that state to appear in the downpass state set, we only need to consider subsets of the observed state set. The fourth subscript indexes the states, thus it must be of size |*S*|.

We can initialize a lookup table *Q_0,{x},{x},x_(n) = *1.0 for each leaf node, *n, *and each state *x* ∈ *S. *All other elements of the *Q* lookup table are set to 0.0 for the leaf nodes. This initialization reflects the fact that there is no opportunity for evolution within the leaf node (the node represents the current state of the OTU). Thus, for any state , *x*, at the leaf node there is a probability of 1 that the observed state set and the downpass state set will both be *{x}*, and every other outcome has a probability of 0.

For an internal node, we can fill in the *Q* lookup table by considering the two possible ways in which a downpass can be formed: via intersection and via union of the downpass state sets of the children. In particular, *Q_s,t,d,a_(n) =* *I*_*s,t,d,a*_ *(n)* + *U_s,t,d,a_(n) *where *I* and *U* use the same subscripting as *Q. *The *I* term conditions on the fact that *d* was formed via an intersection, and the *U* term denotes the probability of the pattern conditional on the fact that *d* was formed by a union in Fitch's algorithm. Because the intersection in Fitch's algorithm does not increase the number of steps assigned to a subtree, we can calculate the *I *term from the combinations of *Q* terms in the children of *n* that have parsimony scores that sum to *s*. To express this mathematically we will introduce several variables. *s_A_* represents the number of parsimony steps contributed by the subtree rooted at the first child, *A(n)*. When we are considering the case of an intersection leading to *d*, we know that the downpass state set of each child must be a superset of *d*. Because a downpass state set of *d* requires at least |*d*| - 1 changes, each child's subtree must contribute at least this number of steps. Thus we have to consider values of s_A_ that range from |*d*|-1 up to *s* + 1 - |*d*|. We will use c_A_ to denote the set of states observed in that subtree, but not in the downpass state set of that subtree; note that *c_A_* must be a subset of *t-d*. Similarly, *g_A_* is the set of states in the downpass state set of *A(n)* but not in *d*; note that *g_A_* must be a subset of *c_A_*. We will use a function abbreviated *C*[...] to refer to the probability of a child subtree displaying a particular class of patterns given the state of the ancestral node *n is* *a*. In particular:



the arguments specify the number of steps in the child's subtree, the observed state set of the child's subtree, the downpass state set of the child, the actual state of the parental node, and *A(n)* for the child node. This function is a similar to portion of the pruning algorithm of Felsenstein. Here *P(i | a, e[A(n)]) *denotes the transition probability, which is the probability of a character state *a* in the ancestor evolving to state *i* in a child across a branch of length *e[A(n)]* (the descendant node,* A(n)*, uniquely specifies an edge in the tree). This notation allows us to express the events of interest in the first child's subtree. We will use a second function, *W*, (defined below), to calculate the probability of the necessary events occurring in the second child's subtree. Taken together, these functions allow us to calculate the *I* term:



as a summation over all possible contributions of the first child's subtree. The *W* function here contributes the probability of evolutionary events in the second that must occur in order to guarantee *s* steps, an observed state set of *t*, and a downpass of *d* in node *n*. The general form is similar to terms seen above:



but the ranges of the summations differs from the previous expressions. Once again, *c_B_* is a subset of *t-d*, but *c_B_* must include all of the states in *t* that were not in *d+c_A_*. This constraint is necessary because the union of the states observed in the first and second subtrees must be equal to *t*. So *c_B_* must be chosen such that *d* ∪ *c_A_* ∪ *c_B_* = *t*. The range of *g_B_* in the summation in *W* must be the subsets of *c_B_*, but it must be restricted to states not found in *g_A_*. This restricted range in the summation is required because if *g_A_* and *g_B_* had a non-empty intersection, these common states would also be found in the ancestor's downpass set (thus the downpass would be larger than the *d* downpass that we aim to calculate).

To calculate the *U_s,t,d,a_(n)* term mentioned above, we must consider the possible outcomes in each subtree. In this case, we rely on the fact that the union of the downpass state sets of the two child nodes must be equal to *d*, and neither downpass can be the empty set:



as before. The new function, *V*, is defined as:



Because the downpass state set of the second child can be found by subtraction, *g_B_=d-g_A_*, this function only entails one summation. *c_B_* is the set of states observed in the second subtree that are not in that *d_B_*. The possible values for *c_B_* in the summation are all of the subsets of *t-g_B_* which include the states in *t - (c_A_ + d)*.

After traversing the entire tree and calculating the *Q* lookup table at the root of the tree, the probability, *X*, of a pattern that belongs to particular class of patterns can be calculated by marginalizing over the root states:



where π denotes the equilibrium state frequency of the model of character evolution and ρ denotes the root node.

## Implementation

MTH has implemented a command-line tool that can report the probabilities of pattern classes for nucleotide data given a fixed tree with branch lengths and values for the numerical parameters of the general time-reversible (GTR) model with invariant sites and gamma-distributed among site rate heterogeneity. The code is available as open source software under the GNU Public License from https://github.com/mtholder/PhyPatClassProb. Compilation depends on the NCL^8^ , BEAGLE^9^ and pytbeaglehon libraries, but a snapshot of the code with dependencies and a build script is posted at http://phylo.bio.ku.edu/software/pattern_class_prob_and_deps.tar.gz.

The implementation reads a tree with branch lengths and takes command line arguments to specify the numerical values for the parameters in the model of character evolution.

**Computational time as a function of number of tips in a tree:**
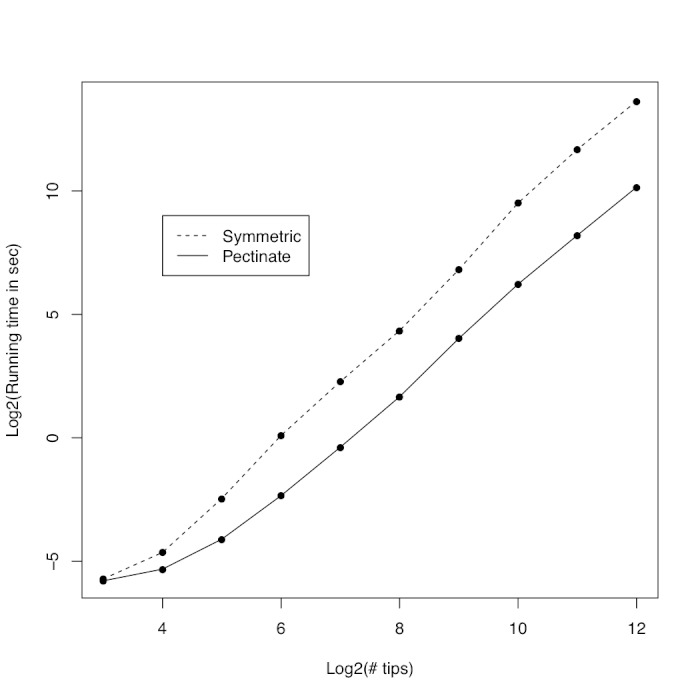

## Validation

As described in the caption of Table 1, we validated our analytical approach by re-analyzing the data set examined by Waddell *et al*. 2 . The counts that Waddell *et al*. found did not deviate significantly from the expected counts based on our algorithm. We converted the expected number of sites per dataset (shown in the Table) to the counts observed by Waddell *et al.* by multiplying the expected number of sites by the size of their simulation (100,000 sites). We compared these observed counts to the expectations from our results using a χ^2 ^goodness-of-fit test (χ^2^ test statistic = 20.9, df = 24) to obtain a *P*-value of 0.64 for the null hypothesis that our algorithm produces the same probabilities that Waddell *et al.*were approximating.

**Table 1: Validation of Data:** Below is a comparison of the expected number of sites in different pattern classes for a tree of 730-base RAG1 sequences from 40 species of mammals. The tree, model, and data are the same as those used by Waddell *et al*. 2, and the expected number of sites from their simulation-based techniques were obtained by summing elements in their table 2 to correspond to the classes of patterns calculated by our algorithm. They estimated the probability of pattern classes by calculating the relative frequency of the patterns based on 100,000 simulated sites.

# Parsimony Steps	# States	Expected number of sites via simulations of Waddell et al.	Expected number of sites calculated by our algorithm
0	1	282	283.21
1	2	110.1	109.00
2	2	48.0	48.60
3	2	25.5	24.67
4	2	12.9	12.59
5	2	6.6	6.30
6	2	3.1	3.09
7-20	2	2.5	2.59
2	3	40.3	40.26
3	3	38.2	38.43
4	3	29.7	29.98
5	3	21.8	21.51
6	3	14.4	14.70
7	3	9.7	9.63
8	3	5.7	5.94
9	3	3.2	3.35
10-26	3	2.8	2.80
3-4	4	16.8	16.43
5	4	11.4	11.39
6	4	11	11.32
7	4	10.3	10.29
8	4	8.2	8.61
9	4	6.5	6.50
10	4	4.3	4.31
11-30	4	4.7	4.51

## Extensions

In addition to conducting marginal tests of models of sequence evolution, other applications require us to calculate the probability of a class of data patterns. Felsenstein^10^ introduces a correction for ascertainment bias which involves calculating the probability of variable patterns. This can be easily done by calculating the probability of the constant patterns and subtracting this from one. More advanced forms of correcting for ascertainment bias are more difficult to correct for. For example, Nylander et al. 11 proposed correcting for the fact that morphological character matrices often lack parsimony-uninformative sites. To implement their correction, one must be able to calculate the probability of the uninformative class of patterns. Exhaustively enumerating these patterns is feasible for binary characters, but the methods that we introduce in this work will allow the usage of this form of correction on data sets that have multi-state characters.

Further work will include producing specialized forms of these algorithms designed for the case in which the rate matrix is symmetric.
